# Mixed States and Suicidality in Bipolar Disorder: Results from the Stanley Foundation Bipolar Treatment Network

**DOI:** 10.1192/j.eurpsy.2023.1202

**Published:** 2023-07-19

**Authors:** M. Macellaro, R. Cafaro, B. Dell’Osso, T. Suppes

**Affiliations:** 1 Department of Mental Health, Department of Biomedical and Clinical Sciences Luigi Sacco, Milan, Italy, University of Milan, Milan, Italy; 2 Department of Psychiatry and Behavioral Sciences, Stanford University, Stanford, United States

## Abstract

**Introduction:**

Mixed states in bipolar disorder (BD) are characterized by the simultaneous occurrence of both manic and depressive features. Growing evidence suggests that they are associated with longer duration of illness, increased relapse risk, and higher prevalence of comorbidities. Suicide risk among BD patients is up to 20–30 times greater than in the general population, although there is not a univocal consensus in whether mixed states should be considered as a high-risk state for suicide.

**Objectives:**

The objective of this study was to identify whether depressive and hypo/manic episodes with mixed features, are associated with more frequent suicidal behavior or ideation when compared to pure hypo/manic and depressive ones. We hypothesized that suicidal ideation or behavior would be more common in those experiencing mixed symptoms. We subsequently also tested whether gender or bipolar subtype moderated the relation between mood and suicidality.

**Methods:**

In a naturalistic study, 903 adult BD outpatients participating in the Stanley Foundation Bipolar Network were followed longitudinally across 14,213 visits for 7 years. The scores at the Inventory of Depressive Symptomatology–Clinician-Rated Version (IDS-C) and at the Young Mania Rating Scale (YMRS), administered at each visit, were used to define the mood episode. Given partial overlap of items between these scales, analyses were also conducted by removing overlapping items. The presence of suicidality was evaluated through the 18th item of the IDS-C (Suicidal Ideation).

**Results:**

During the observation period, up to 60.7% of subjects had at least one visit with suicidal ideation or behavior, broadly defined as a score ≥ 1 at the 18th item of the IDS-C. The distribution of suicidality among visits in different mood states is reported in Figure 1 (N° of visits in which suicidality was detected by mood state at the time of visit). Depressive symptoms were associated with suicidal ideation and behavior either during mixed depression and hypo/mania (p < 0.0001). Hypomanic or manic symptoms appeared to be related to suicidality only during hypo/mania, either pure or mixed (p 0.007). When overlapping items between the two psychometric scales were removed results seemed to confirm these hypotheses. Moreover, male gender appeared to have a protective role against suicidal ideation when hypo/manic (p 0.002).

**Image:**

**Figure:**
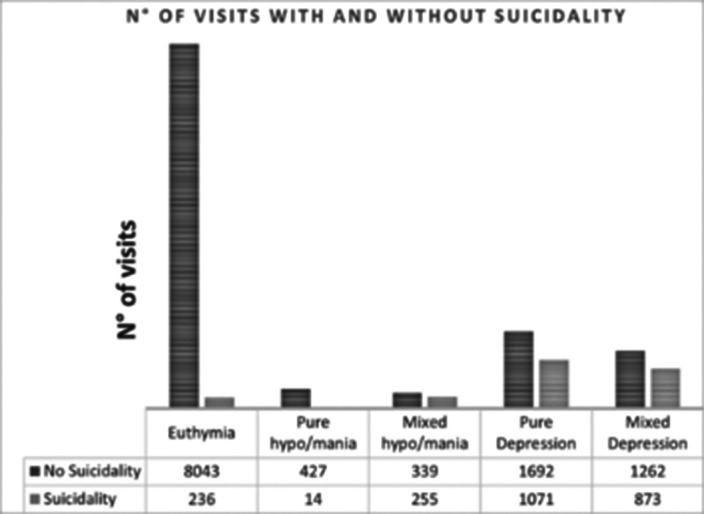

**Conclusions:**

The primary role of depressive symptoms in predicting suicidal risk was confirmed by our results, which however suggest that mixed symptoms relate to suicidal ideation and behavior more intensely than pure hypo/manic symptoms. More importantly, hypo/manic symptoms did not appear to have a protective role during mixed episodes. Future studies considering also deaths by suicide are required in order to better clarify the role of mixity on suicidality.

**Disclosure of Interest:**

None Declared

